# Improvement of Metastatic Colorectal Cancer Patient Survival: Single Institution Experience

**DOI:** 10.3390/cancers11030369

**Published:** 2019-03-15

**Authors:** Elisabetta Fenocchio, Federica Colombi, Maria Grazia Calella, Roberto Filippi, Ilaria Depetris, Giovanna Chilà, Pasquale Lombardi, Donatella Marino, Celeste Cagnazzo, Renato Ferraris, Marco Vaira, Massimo Aglietta, Francesco Leone

**Affiliations:** 1 Department of Oncology, University of Turin Medical School, Corso Dogliotti, 38, 10126 Turin, Italy; mariagcalella@gmail.com (M.G.C.); roberto.filippi@ircc.it (R.F.); depetris.ila@gmail.com (I.D.); giovanna.chila@ircc.it (G.C.); pasquale.lombardi@ircc.it (P.L.); massimo.aglietta@unito.it (M.A.); francesco.leone@ircc.it (F.L.); 2 Department of Medical Oncology, Candiolo Cancer Institute, FPO—IRCCS—Str. Prov.le 142, km 3.95, 10060 Candiolo (TO), Italy; federica.colombi@gmail.com (F.C.); donatella.marinomd@gmail.com (D.M.); renato.ferraris@ircc.it (R.F.); 3 Unità di Ricerca e Sviluppo Clinico S.C. Oncoematologia Pediatrica—AOU Città della Salute e della Scienza, Presidio Ospedaliero Infantile Regina Margherita, 10126 Turin, Italy; celeste.cagnazzo@gmail.com; 4 Dipartimento di Scienze della Sanità Pubblica e Pediatriche—Università degli Studi di Torino, 10126 Turin, Italy; 5 Department of Surgery, Candiolo Cancer Institute, FPO—IRCCS—Str. Prov.le 142, km 3.95, 10060 Candiolo (TO), Italy; marco.vaira@ircc.it

**Keywords:** metastatic colorectal cancer, overall survival, integrated approach, chemotherapy, surgery, targeted agents

## Abstract

The survival rates of patients with metastatic colorectal cancer (mCRC) have improved in recent years. We analysed the survival of mCRC patients followed at a single institution over the last 17 years. We retrospectively collected data from 899 mCRC patients treated from 2001 to 2016. Patients were divided into two groups based on the year of diagnosis: Cohort A (2001–2006) and Cohort B (2007–2014). A total of 788 patients were analysed. The median survival of the whole population was 32.0 months with a significant difference between Cohort A and B (29.2 vs. 33.5 months; *p* = 0.041). Surgical procedures significantly increased in Cohort B, however, no significant changes in survival were observed in patients undergoing surgery (58.9 months Cohort A vs. 58.2 months Cohort B, *p* = 0.822). Similarly, we did not demonstrate survival improvement in patients treated with systemic therapy alone (18.9 months Cohort A vs. 20.7 months Cohort B; *p* = 0.948). At the multivariate analysis, right-sided primary and synchronous metastatic tumour were found to be independent unfavorable prognostic factors. Improvements of mCRC patient survival might relate to integrated approach, with more patients undergoing extra-hepatic surgery. The medical approach seems to have had a more favourable impact on subgroups characterized by a worse prognosis.

## 1. Introduction

Colorectal cancer (CRC) represents the second most commonly diagnosed cancer in Europe, with 215,000 deaths in 2012 [1,2].

Over the last two decades, the outcome of patients with metastatic CRC (mCRC) has significantly improved, reaching a median overall survival (mOS) of around 30 months, more than double that 20 years ago [3,4].

This advancement may result from the contribution of a more aggressive systemic approach—including three-drug chemotherapy (CT) schemes and CT with targeted therapy combinations—together with a substantial change in surgical indication [5,6,7].

In our study, we investigated the possible influence of tumour characteristics as well as the changes in treatment practice through the years, on the evolution of survival of mCRC patients.

## 2. Results

### 2.1. Demographics

A total of 1702 patients with CRC were treated at our institution from 1999 to 2016. 

Patients with localized CRC who received adjuvant CT only and those with mCRC who had never been treated with chemotherapy were excluded from the analysis.

Overall, 899 patients were available for the analysis, 788 of whom displayed a sufficient completeness of treatment and outcome data to be included in the survival analysis.

The median age at diagnosis was 62.58 years. 

All main disease and treatment characteristics are summarized in Table 1.

### 2.2. Treatment

Considering the whole population, patients who underwent surgery significantly increased (42% in Cohort A vs. 58% in Cohort B; *p* < 0.009). A total of 203 patients only received hepatic surgery (93 in Cohort A vs. 110 in Cohort B). The details of surgery in the two cohorts are listed in Table 2 and Table 3.

Eleven patients in Cohort B received hypertermic intraperitoneal chemotherapy (HIPEC) and/or pressurised intraperitoneal aerosol chemotherapy (PIPAC) procedures.

In the first line setting, two-drug CT containing oxaliplatin was the main choice in both cohorts (65.1% in Cohort A and 66.3% in Cohort B). Conversely, the use of irinotecan-based doublets was significantly higher in Cohort B (20.7%) compared with Cohort A (13.6%) in this setting, while it became the predominant CT adopted in both cohorts in the second line setting (54.3% in Cohort A and 56.7 % in Cohort B). Only 1% of patients received FOLFOXIRI regimen in Cohort B, especially in the first line setting. Conversely, the number of patients receiving monotherapy was significantly lower in all the treatment lines in more recent years. 

Differences were also noted in the administration of anti-EGFR and anti-VEGF antibodies and an increase between the two cohorts in both the first and the second line settings was observed. Details of treatment administration in the two cohorts are presented in Table 4.

### 2.3. Survival Analysis

The survival analysis was carried out on 788 patients (46.3% in Cohort A and 53.7% in Cohort B). The median follow-up time was 24.7 months (23.8 months in Cohort A and 25.6 months in Cohort B).

**Figure 1:**
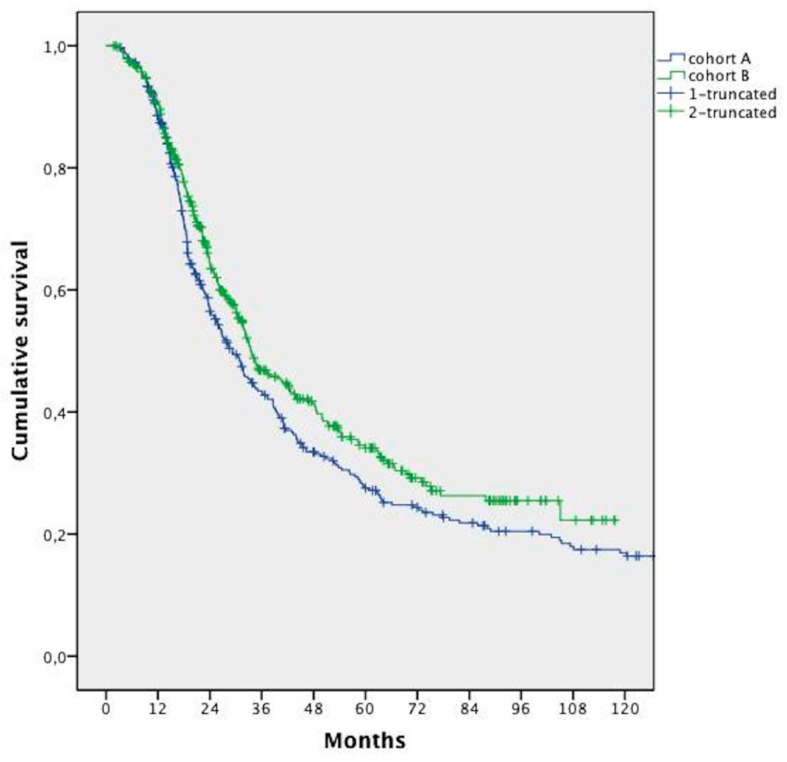
Median overall survival analysis in the two cohorts.

The mOS was 32.0 months (95% confidence interval (CI); 28.8 to 35.3 months). Patients’ survival in Cohort B was significantly longer compared with that in Cohort A (median 33.5 months vs. 29.2 months, respectively, HR 0.832; 95% CI 0.697–0.992; *p* = 0.041) (Figure 1).

Among patients included in the survival analysis, 456 underwent resection of hepatic and/or other metastatic sites, with a larger proportion of surgical patients in Cohort B (58% vs. 42%; *p* < 0.009). While hepatic resection rates remained similar through the years (20.3% Cohort A vs. 24.1% Cohort B), resection of metastases at other sites with or without hepatic surgery was performed with increasing frequency (21.4% Cohort A vs. 33.9% Cohort B; *p* < 0.005). In particular, the largest increase was in peritoneal surgery.

**Figure 2:**
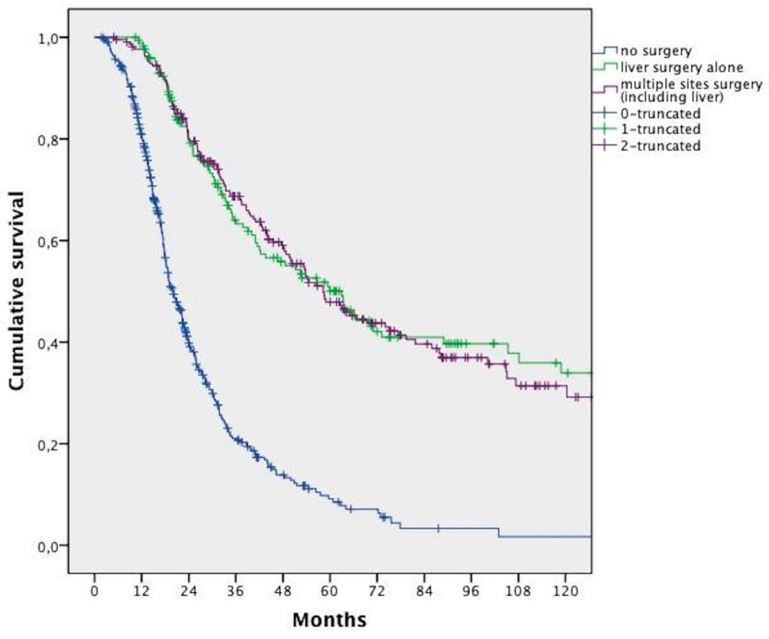
Overall survival analysis of patients who underwent liver surgery vs. surgery of multiple sites (including liver) vs. no surgery.

In the whole population, surgery in combination with CT allowed a significantly longer survival when compared with CT alone (median 58.5 months vs. 20.1 months, respectively, HR 0.262; 95% CI 0.216–0.316; *p* < 0.0001). This advantage was maintained even when distinguishing between liver surgery and surgery of other sites, with or without hepatic surgery (Figure 2).

**Figure 3:**
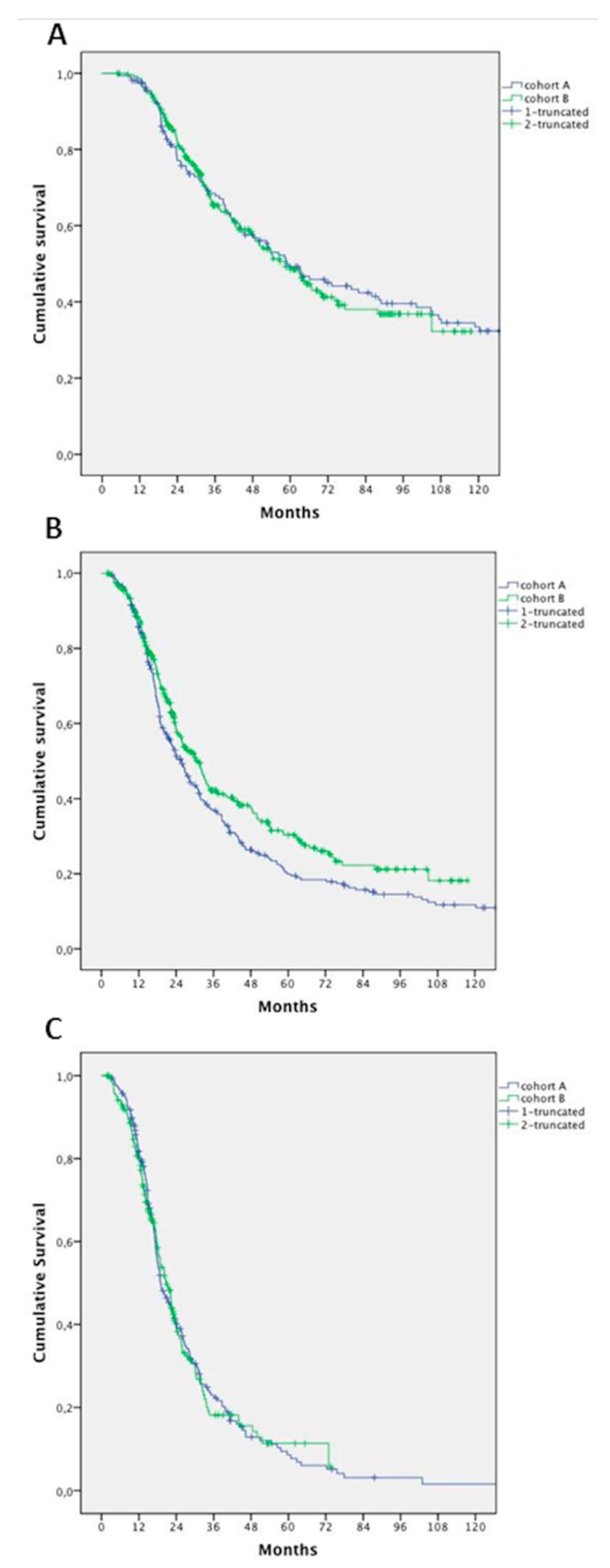
Survival analysis according to treatment in Cohort A and B: (**A**) patients undergoing systemic treatment and surgery; (**B**) patients undergoing systemic treatment and surgery, excluding patients who had hepatic resection alone; and (**C**) patients undergoing exclusively systemic treatment.

No differences in survival of patients who underwent surgery—in addition to a systemic treatment—were detected between cohorts (median 58.9 months vs. 58.2 months, HR 1.033; 95% CI, 0.779–1.369; *p* = 0.822) (Figure 3A).

After excluding patients who had hepatic resection alone, we found a significant improvement in survival in patients in Cohort B (25.7 months in Cohort A vs. 30.4 months in Cohort B; HR 0.796; 95% CI 0.656–0.967; *p* = 0.021) (Figure 3B).

We failed to demonstrate an improvement in OS in patients treated with CT alone (with or without targeted agents): mOS 18.9 months in Cohort A versus 20.7 months in Cohort B (HR 1.0; 95% CI 0.799–1.271; *p* = 0.948) (Figure 3C).

### 2.4. Prognostic Factors

The prognostic role of clinical characteristics was analysed by uni- and multivariate analysis (Table 5).

At the multivariate analysis, a right-sided primary tumour and synchronous metastatic disease were found to be independent unfavorable prognostic factors. All these characteristics had a well-balanced distribution between cohorts (Table 6).

**Figure 4:**
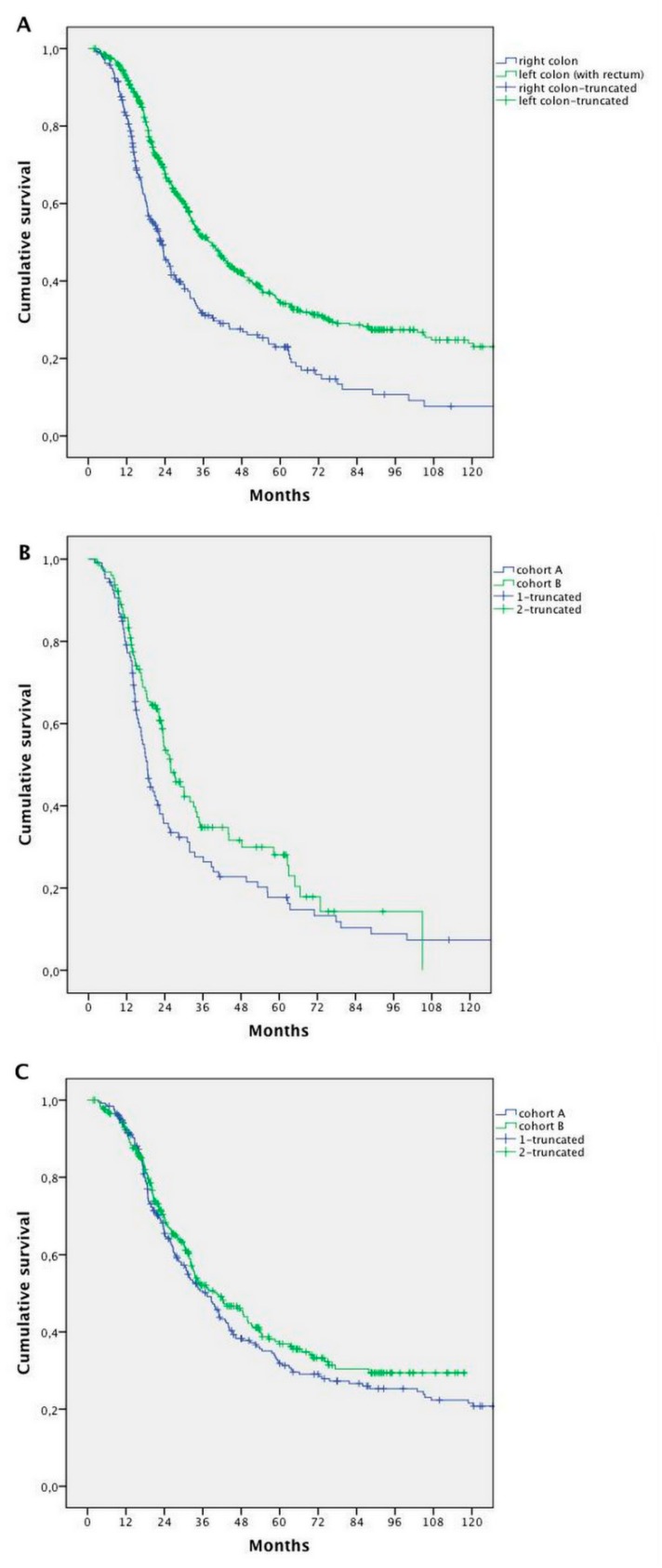
Survival analysis according to the primary tumor site: (**A**) survival analysis of patients with right-sided primary tumor vs. left-sided primary tumor (whole population); (**B**) survival analysis for patients with right-sided primary tumor in Cohort A vs. Cohort B; and (**C**) survival analysis for patients with left-sided primary tumor in Cohort A vs. Cohort B.

In the whole population, a worse prognosis was observed for patients with right-sided compared with left-sided primary tumours—mOS 22.9 versus 38.6 months, respectively (HR 1.709; 95% CI 1.427–2.047; *p* < 0.0001) (Figure 4A).

Survival seemed to increase in both left- and right-sided patients in Cohort B compared with Cohort A. However, the difference was statistically significant for right-sided tumours only (18.5 months Cohort A vs. 25.8 months Cohort B, HR 0.738; 95% CI 0.546–0.998; *p* = 0.044), without changes in the left-sided ones (34.5 months Cohort A vs. 37.5 months Cohort B; HR 0.883; 95% CI 0.638–1.223; *p* = 0.455) (Figure 4B,C).

After excluding surgery, a significant improvement was achieved in patients treated with systemic therapy only (15.8 months Cohort A vs. 22.1 months Cohort B, *p* = 0.041), while mOS in resected patients remained stable over time for right-sided tumours (49.6 months Cohort A vs. 48.2 months Cohort B, *p* = 0.736).

**Figure 5:**
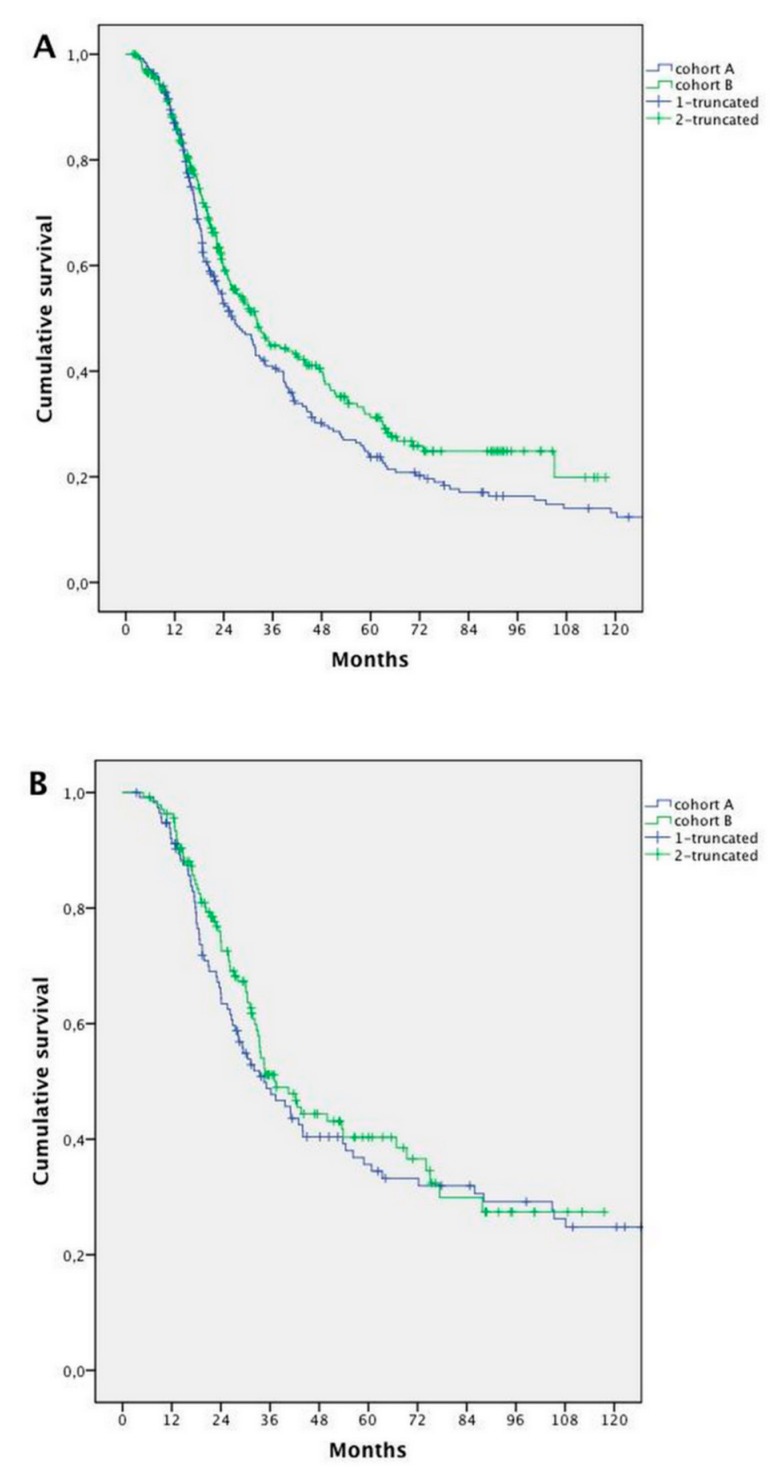
Survival analysis in synchronous vs. metachronous metastases: (**A**) survival analysis of patients with synchronous metastases in Cohort A vs. Cohort B; and (**B**) survival analysis in the two Cohorts for patients with metachronous metastases in Cohort A vs. Cohort B.

Median survival was 36.2 months for patients with metachronous disease and 30.0 months for patients with synchronous disease (HR 0.718; 95% CI 0.592–0.870; *p* = 0.001). Patients with synchronous disease trended to show better rates of survival in Cohort B (median 26.0 months Cohort A vs. 31.9 months Cohort B; HR 0.814; 95% CI 0.660–1.005; *p* = 0.05), while no significant improvement was recorded in metachronous disease (34.5 months Cohort A vs. 37.5 months Cohort B; HR 0.883; 95% CI 0.638–1.223; *p* = 0.455) (Figure 5)

## 3. Discussion

Recent studies in mCRC have shown that new drug combinations allow patients to achieve mOS rates of around 30 months or more [5,6,7]. However, these results might overestimate real world data because clinical study subjects generally represent a selected population with better prognostic characteristics compared with the general population. When currently used combinations were published in the early 2000s [8,9,10], it became evident that mOS of mCRC patients could reach 21 to 24 months with CT and monoclonal antibodies (mAb). However, any comparison of those results with more recent data would be hindered by a number of biases.

In recent years, widespread awareness of the potential for cure has led to intensified treatment of mCRC, including multidisciplinary discussion and multimodality approach. Furthermore, molecular selection has gained an unquestionable role in exploiting EGFR inhibition and, in keeping with the rules in force, anti-EGFRs have also been introduced in first line treatments.

In this study, we looked into whether changes in practice might have influenced survival rates in an unselected population. The patients were treated at a single institute according to current guidelines and were retrospectively analysed. Two periods, for Cohort A until 2006, and for Cohort B from 2007 onwards, were compared.

The results of our analyses revealed that patients in Cohort B lived longer than patients in Cohort A. Kopetz et al. [11] demonstrated that in patients diagnosed from 1998 to 2004, improvements in outcomes were mainly associated with an increase in surgery, whereas in the period from 2004 to 2006, the rise was related to advances in medical therapy. In our series, the percentage of patients who underwent surgery increased from 42% in Cohort A to 48% in Cohort B, while liver resection was stable at around 22%. This percentage was nearly the same as that of Kopetz et al.; however, they did not analyse extra-hepatic surgery data. A previous report from a multicenter study, including our own, highlighted the role of surgery of lung metastases in improving survival [12]. Moreover, surgical management of mCRC has developed in recent years to include cytoreductive surgery of peritoneal metastases and HIPEC. These approaches are now evolving as new standards of treatment in highly specialized centers [13]. In our study, the percentage of patients who underwent extra-hepatic surgery increased from 21% in Cohort A to 34% in Cohort B and the survival curves of these patients were similar to those of patients who had liver surgery alone. It is of note that, whereas the survival rates of patients receiving liver surgery alone did not differ between cohorts, there was a significant increase of survival in the subgroup of patients who had extra-hepatic surgery in recent years.

The main change observed in medical treatment practice, along with the introduction of mAb, was the adoption in the first-line of a two-drug in a larger percentage of patients in Cohort B, leaving the monotherapy to a subgroup of patients with poor prognosis. This reflects the updating of international guidelines, which previously considered asymptomatic patients with low burden disease as suitable for sequential schedules of monotherapy [4]. More recently, a general strategy has evolved to consider a CT doublet with mAb as an appropriated choice, where the goal is disease control for patients for whom intensive treatment is not necessary [14]. Even in unfit patients, capecitabine plus bevacizumab or a reduced-dose doublet of cytotoxics or anti-EGFR-containing therapy can be considered [3]. 

Despite the evolution of medical strategies, if we compare survival rates of patients treated with CT alone, the increase of survival between the two cohorts was not significant in the whole population. However, considering patients with right-sided primary tumour, an independent negative prognostic factor, the survival of patients in Cohort B was significantly longer than in Cohort A. The prognostic role of tumour sideness has been known for a long time, however, recent studies have shed light on the biological causes of these differences [15,16]. By separately analyzing patients with right-sided or left-sided tumours, we found that while survival increased in both groups, the difference was statistically significant in the right-sided group only. Furthermore, considering synchronous and metachronous metastatic groups, the improvement of survival in Cohort B was more evident for patients with synchronous metastatic disease.

## 4. Materials and Methods

### 4.1. Study Design

The study is a retrospective, single institution experience, conducted on mCRC patients attending the Candiolo Cancer Institute from 1999 to 2016. All patients were treated, after informed consent was signed, according to institutional and national guidelines. 

The aim of this study was to describe the evolution of survival of mCRC patients followed at a single institution over the past 17 years. All the patients were screened using the International Classification of Diseases 9th revision Clinical modification (ICD-9-CM) codes for colon and rectal cancer. Only patients receiving at least one course of therapy for mCRC were included. Both metachronous and synchronous metastatic disease presentations were taken into account. Metastatic disease was considered synchronous when diagnosed within six months after the initial diagnosis of CRC. For each patient, demographic and clinicopathological data were collected. We defined as a line of treatment all the therapies administered between the evidence of disease progression until further progression, even in the case of multimodality treatment.

All the descriptive analyses of the patients’ characteristics included patients treated between January 1999 and November 2016. However, in order to minimize the risk of bias related to a small group of patients treated before 2001 (the date of the setting up of a multidisciplinary team for mCRC treatment) and to obtain a minimum follow-up period of two years, the survival analysis did not include patients diagnosed before 1 January 2001 or after 31 December 2014. 

Overall survival was calculated from the date of diagnosis of metastatic disease until death, as notified by the registry office, or censored at the last follow-up visit. The median follow-up period was calculated on the basis of median survival without considering life status. In order to detect survival changes, patients were divided into two groups according to the year of metastatic disease diagnosis: Cohort A (between 2001 and 2006) and Cohort B (between 2007 and 2014). The cut off point was set at 2006 as this date coincided with the introduction of the molecular targeted agents in clinical practice.

### 4.2. Statistical Analysis

SPSS for Windows 20.0 (IBM SPSS, Chicago, IL, USA) was used for all statistical analyses. The differences between proportions were evaluated by the chi-square test with Yates correction, when appropriate. The Cox proportional hazards regression model was used and survival curves were plotted using Kaplan–Meier and compared by log-rank test. Overall survival was calculated from the date of diagnosis of metastatic disease until death, or censored at the last follow-up visit.

### 4.3. Ethics Approval and Consent to Participate

The study was performed in accordance with the Declaration of Helsinki. The development and the publication of this report was approved by the Institutional Review Board. The Medical Ethical Committee of the IRCCS Candiolo Cancer Institute confirmed that formal ethical approval was not required given the retrospective and observational nature of the study.

## 5. Conclusions

We realise that our study is limited by various biases related to the retrospective nature of our analyses. However, our results suggest that in current clinical practice, unless patients are classified as unfit for therapy, the therapeutic strategy is moving towards intensive treatment where at least two cytotoxic therapies are combined together with biological agents, and a multimodal approach in which surgery of metastatic sites is considered feasible. This approach seems appropriate to increase patient survival. In particular, it is likely that poor prognostic subgroups of mCRC patients would benefit from an integration of medical and surgical treatments in a ‘*continuum* of care’ strategy.

## Figures and Tables

**Table 1:** Population characteristics.

Characteristics		Value	
		*N*	%
**Primary tumour site**	Right Colon	266	29.6%
	Left Colon (with rectum)	622	69.1%
	Unknown	11	1.4%
**Histological grade**	G1–2	289	32.1%
	G3–4	234	26%
	Not determined	374	41.6%
**Chronology of metastases**	Metachronous	298	33.1%
	Synchronous	601	66.8%
**Metastatic sites at diagnosis**	Liver	605	67.2%
	Lung	216	24%
	Lymph nodes	151	16.8%
	Peritoneum	141	15.7%
	Ovary/Uterus	34	3.8%
	Bone	11	1.2%
	CNS	3	0.3%
**Molecular Characteristics**			
**KRAS**	Wild type	213	23.7%
	Mutated	156	17.3%
	Not determined	530	58.9%
**NRAS**	Wild type	99	11%
	Mutated	8	0.9%
	Not determined	792	88%
**BRAF**	Wild type	140	15.6%
	Mutated	13	1.4%
	Not determined	746	82.9%
**PI3KCA**	Wild type	56	6.2%
	Mutated	10	1.1%
	Not determined	833	92.6%
**MSI**	MSS	167	18.6%
	MSI-H	14	1.6%
	Not determined	718	79.8%
**Treatment Characteristics**			
**Chemotherapy**		899	100%
**Surgery of metastases**		456	50.7%
**Radiotherapy (palliative RT excluded)**		61	6.8%
**Locoregional treatments**		18	2%
**HIPEC/PIPAC**		11	1.2%

Abbreviations: G = grade; CNS = central nervous system; MSS = microsatellite stable; MSI = microsatellite instable; RT = radiotherapy; HIPEC = hypertermic intraperitoneal chemotherapy; PIPAC = pressurised intraperitoneal aerosol chemotherapy.

**Table 2:** Liver surgery vs. surgery of other sites.

Type of Surgery	Surgery of Metastases		Extra-Hepatic Surgery		Liver Surgery Alone	
	*N*	%	*N*	%	*N*	%
**Cohort A**	191	42%	98	21.4%	93	20.3%
**Cohort B**	265	58%	155	33.9%	110	24.1%
**Total**	456	50.7%	253	28%	203	22.6%
***p-*** **value**	<0.009		<0.005		<0.91	

**Table 3:** Surgery sites and distribution.

Surgery		Patients (*N*)		*p*-Value (Chi Square)
**1st Line**		Cohort A	Cohort B	
**Surgery**	NO	235	241	0.041
	YES	180	243	-
**Sites**	liver	118	124	0.071
	lymph nodes	1	10	-
	spleen	1	0	-
	ovary	7	14	-
	lung	13	27	-
	pelvis	15	15	-
	peritoneum	10	20	-
	CNS	0	1	-
	other sites	0	2	-
	adrenal gland	0	1	-
	multiple sites	15	29	-
**2nd Line**		Cohort A	Cohort B	
**Surgery**	NO	352	409	0.896
	YES	63	75	-
**Sites**	liver	20	27	0.489
	lymph nodes	3	2	-
	spleen	0	1	-
	ovary	2	5	-
	pelvis	8	4	-
	peritoneum	6	5	-
	lung	16	21	-
	CNS	4	1	-
	other sites	1	1	-
	adrenal gland	0	1	-
	multiple sites	3	6	-
**3rd Line**				
**Surgery**	NO	393	451	0.344
	YES	22	33	-
**Sites**	liver	5	9	0.357
	lymph nodes	0	1	-
	ovary	1	0	-
	pelvis	3	0	-
	peritoneum	2	5	-
	lung	6	8	-
	CNS	1	4	-
	adrenal gland	0	1	-
	multiple sites	4	4	-
**4th Line**				
**Surgery**	NO	411	480	0.827
	YES	4	4	-
**Sites**	liver	1	1	0.638
	pelvis	0	1	-
	lung	0	1	-
	multiple sites	1	0	-
	CNS	2	1	-

Abbreviation: CNS = central nervous system.

**Table 4:** Treatment characteristics.

Treatment Characteristics		1st Line		2nd Line		3rd Line		4th Line	
		Cohort A (415 pts)	Cohort B (484 pts)	Cohort A (372 pts)	Cohort B (386 pts)	Cohort A (282 pts)	Cohort B (284 pts)	Cohort A (168 pts)	Cohort B (161 pts)
		*N* (%) ^a^	*N* (%) ^a^	*N* (%) ^a^	*N* (%) ^a^	*N* (%) ^a^	*N* (%) ^a^	*N* (%) ^a^	*N* (%) ^a^
**Chemo therapy and targeted therapy**	FOLFOXIRI	none	3 (1.2%)	none	none	none	none	none	1 (1.1%)
	Oxaliplatin doublet	152 (65.1%)	160 (66.3%)	60 (22.4%)	49 (18.5%)	40 (21.7%)	56 (32%)	35 (32.7%)	22 (24.4%)
	Irinotecan doublet	32 (13.6%)	50 (20.7%)	145 (54.3%)	151 (56.7%)	37 (20.1%)	39 (22.3%)	8 (7.5%)	20 (22.2%)
	Monotherapy ^a^	51 (21.7%)	27 (11.2%)	62 (23.3%)	66 (24.8%)	106 (57.6%)	71 (40.6%)	64 (59.8%)	38 (42.2%)
	*p* value (chi square)	<0.0001		0.489		0.001		<0.0001	
	Anti-EGFR ^b^	none	24 (23.7%) ^c^	8 (10.2%) ^c^	31 (24.8%) ^c^	41 (59.4%) ^c^	52 (53.6%) ^c^	13 (27.1%) ^c^	20 (40.1%) ^c^
	Anti-VEGF ^b^	6 (5.6%)	69 (76.6%)	2 (0.7%)	55 (20.6%)	4 (2.1%)	21 (12%)	6 (5.6%)	4 (4.4%)
	*p* value (chi square)	0.004		<0.0001		<0.0001		0.166	
	Total *	**235** **(56.6%)**	**241** **(49.8%)**	**267** **(71.8%)**	**266** **(68.9%)**	**184** **(65.2%)**	**175** **(61.6%)**	**107** **(63.9%)**	**90** **(55.9%)**
**Chemo therapy + surgery**	FOLFOXIRI	none	2 (0.8%)	none	none	none	none	none	none
	Oxaliplatin doublet	110 (67.9%)	142 (63.6%)	15 (38.4%)	11 (22.9%)	3 (27.3%)	5 (27.7%)	none	1 (100%)
	Irinotecan doublet	14 (8.6%)	52 (23.3%)	20 (51.2%)	23 (47.9%)	3 (27.3%)	7 (39%)	none	none
	Monotherapy ^a^	38 (23.4%)	27 (12.1%)	4 (10.2%)	14 (29.1%)	5 (45.4%)	6 (33.3%)	2 (100%)	none
	*p* value (chi square)	<0.0001		0.064		0.766		0.083	
	Anti-EGFR ^b^	1 (2.4%) ^c^	10 (9.7%) ^c^	none	7 (22.5%) ^c^	3 (42.8%) ^c^	6 (40.0%) ^c^	none	none
	Anti-VEGF ^b^	1 (0.6%)	49 (21.9%)	1 (2.5%)	6 (22.2%)	1 (9%)	none	none	none
	*p* value (chi square)	<0.0001		0.007		0.422		-	
	Total *	**162** **(39%)**	**223** **(46.1%)**	**39** **(10.5%)**	**48** **(12.4%)**	**11** **(3.9%)**	**18** **(6.3%)**	**2** **(1.2%)**	**1** **(0.6%)**
**Surgery alone**		18 (4.3%)	20 (4.1%)	24 (6.4%)	27 (9.4%)	11 (3.9%)	15 (5.3%)	2 (1.2%)	3 (1.8%)
**Not treated**		none	none	42 (11.2%)	45 (11.6%)	76 (26.9%)	76 (26.7%)	57 (33.9%)	67 (41.6%)

* total of patients treated in the subgroup in this line; ^a^ percentage calculated relative to the number of patients treated in the subgroup for that line of therapy; ^b^ monoclonal antibody associated or not to chemotherapy; ^c^ percentage calculated relative to the number RAS or KRAS exon2 wild type or EGFR+ patients can be treated in this cohort and in this line.

**Table 5:** Univariate and multivariate analysis. CI—confidence interval.

Variable	Number of Patients (A vs. B)	Median Overall Survival (Months)	Univariate		Multivariate	
			HR (95% CI)	*p-*Value	HR (95% CI)	*p-*Value
**Primary tumour site** **(Right vs. Left)**	236 vs. 542	22.9 vs. 38.6	1.774 (1.470–2.140	<0.0001	0.596 (0.491–0.723)	<0.0001
**Histological grade** **(G1–G2 vs. G3–G4)**	246 vs. 209	34.5 vs. 33.0	1.058 (0.839–1.334)	0.634	ns	ns
**T status** **(T1–T2 vs. T3–T4)**	51 vs. 554	43.9 vs. 38.6	1.293 (0.889–1.878)	0.178	ns	ns
**N status** **(N0 vs. N+)**	191 vs. 406	43.0 vs. 33.3	1.344 (1.076–1.679)	0.009	ns	ns
**Chronology of mts** **(Synchronous vs. Metachronous)**	536 vs. 252	30.0 vs. 36.2	0.718 (0.592–0.870)	0.001	0.764 (0.628–0.929)	0.007
**KRAS Mutational Status** **(Wild Type vs. Mutated)**	185 vs. 122	42.0 vs. 34.4	1.079 (0.793–1.469)	0.626	ns	ns
**BRAF Mutational Status** **(Wild type vs. Mutated)**	103 vs. 10	49.9 vs. 102.9	0.696 (0.251–1.931)	0.486	ns	ns
**Liver mts at diagnosis** **(YES vs. NO)**	530 vs. 258	30.7 vs. 34.5	1.142 (0.946–1.378)	0.167	ns	ns
**Lung mts at diagnosis** **(YES vs. NO)**	194 vs. 594	30.0 vs. 32.7	1.089 (0.888–1.335)	0.412	ns	ns
**Lymph nodes mts at diagnosis** **(YES vs. NO)**	141 vs. 647	23.3 vs. 34.2	1.617 (1.296–2.018)	< 0.0001	1.533 (1.225–1.919)	<0.0001
**Peritoneal mts at diagnosis (YES vs. NO)**	121 vs. 667	31.0 vs. 32.7	1.286 (1.009–1.638)	0.048	ns	ns
**Ovarian mts at diagnosis (YES vs. NO)**	32 vs. 756	42.4 vs. 31.8	0.766 (0.478–1.226)	0.267	ns	ns
**Bone mts at diagnosis** **(YES vs. NO)**	8 vs. 780	9.52 vs. 32.2	4.370 (2.165–8.820)	<0.0001	4.427 (2.181–8.984)	<0.0001
**CNS mts at diagnosis** **(YES vs. NO)**	3 vs. 785	20.3 vs. 32.1	2.731 (0.876–8.513)	0.083	ns	ns
**Bone mts** **(YES vs. NO)**	80 vs. 707	28.3 vs. 32.2	1.043 (0.783–1.390)	0.774	ns	ns
**CNS mts** **(YES vs. NO)**	41 vs. 746	30.7 vs. 32.1	1.137 (0.776–1.664)	0.510	ns	ns

Abbreviations: CNS = central nervous system; mts = metastases; ns = not significant.

**Table 6:** Unfavourable prognostic factors distribution.

Prognostic Factors	Cohort A		Cohort B		*p*-Value
	*N*	%	*N*	%	
**Right Colon**	107	29.3%	129	30.5%	0.766
**Left Colon**	252	69.0%	290	68.6%	
**Synchronous mts**	250	68.5%	286	67.6%	0.791
**Metachronous mts**	115	31.5%	137	32.4%	
**Lymph nodes mts**	59	16.2%	82	19.4%	0.239
**Bone mts**	3	0.8%	5	1.2%	0.615

Abbreviation: mts = metastases.
